# Effect of the COVID-19 pandemic on emergency department utilization of computed tomography scans of appendicitis and diverticulitis

**DOI:** 10.1007/s10140-023-02125-w

**Published:** 2023-03-29

**Authors:** Brandon Wayne Collins, Andrew Robart, Evan James Lockyer, Nicholas A. Fairbridge, Tara Rector, Angus Hartery

**Affiliations:** Faculty of Medicine, Memorial University, St. John’s, Canada

**Keywords:** COVID-19, Imaging utilization, Abdominal pathologies, Emergency radiology, Diverticulitis, Appendicitis

## Abstract

**Purpose:**

Investigating the effect of the COVID-19 lockdown on adult patient visits, computed tomography (CT) abdominal scans, and presentations of appendicitis and diverticulitis, to emergency departments (ED) in St. John’s NL.

**Methods:**

A retrospective quantitative analysis was applied, using ED visits and Canadian Triage and Acuity Scale (CTAS) scores. *mPower* (Nuance Communications, UK) identified CT abdominal scan reports, which were categorized into (1) normal/other, (2) appendicitis, or (3) diverticulitis. Time intervals included pre-lockdown (January–February), lockdown (March–June), and post-lockdown (July–August). Data from 2018 to 2019 (January–August) were used to generate expected patient volumes for 2020, and pre- and post-lockdown were included to control for other variables outside the lockdown.

**Results:**

Chi-squared goodness of fit tested for deviations from predicted means for 2018–2019. Compared to expectations, daily ED visits from January to August 2020 showed a significant (*p* < 0.001) decrease in patient volumes independent of gender, age, and CTAS scores. During and post-lockdown, CT abdominal scans did not drop in proportion to patient volume. Appendicitis presentations remained indifferent to lockdown, while diverticulitis presentations appeared to wane, with no difference in combined complicated cases in comparison to what was expected.

**Conclusion:**

During lockdown, significantly fewer patients presented to the ED. The proportion of ordered CT abdominal scans increased significantly per person seen, without change in CTAS scores. Considering combined pathology cases increased during the lockdown, ED physicians were warranted in increasing abdominal imaging as patients did not avoid the ED. This may have resulted from a change in clinical practice where the uncertainty of COVID-19 increased CT scan usage.

## Introduction

Severe acute respiratory syndrome coronavirus 2 (SARS-CoV-2) originated within the city of Wuhan, China, in late December 2019 [1]. SARS-CoV-2 is the virus responsible for the coronavirus disease 2019 (COVID-19) [2], which since its discovery has caused significant implications for the world’s economic, social, and healthcare systems [3, 4]. The original COVID-19 was known to spread from the respiratory tract via droplets, secretions, and direct contact [5]. Although most individuals with COVID-19 present with mild flu-like symptoms, several patients become critically ill, developing respiratory distress syndrome. This includes respiratory failure, multiple organ failure, or even death [6]. Due to these implications, the World Health Organization declared COVID-19 a pandemic on March 11th, 2020 [7]. Since the discovery of COVID-19, the virus has infected over 639,000,000 people, with over 6,600,000 deaths worldwide as of November 2022 [8].

To help mitigate the negative effects of COVID-19, many countries implemented national containment responses such as curfews, lockdowns, and stay-at-home orders. Consequently, these precautions have been shown to decrease the daily total of positive cases [9]. Newfoundland and Labrador (NL) was not spared from COVID-19, with an initial news release concerning COVID-19 being made on March 6th, 2020, and the first presumptive case on March 14th, 2020 [10]. Due to these circumstances, on March 15th, 2020, restrictions were implemented by public health NL regarding Regional Health Authority Facilities, and a public health emergency was declared on March 18th, 2020 [11]. This initial COVID-19 lockdown continued until June 24th, 2020, when a controlled reopening was announced [12]. The public health actions were swift, limiting the number of COVID-19 cases in the province to 261 over the 3-month period [13]. Thus, by implementing restrictions on the public, COVID-19 was strongly contained within NL.

Although lockdowns and precautions decreased cases [9], there has been a substantial amount of sequelae on non-COVID-19 related issues within healthcare systems. For example, multiple countries have shown a reduced amount of emergency department (ED) visits during the pandemic [14–22]. This reduction in ED visits coincided with less patients presenting to the ED with myocardial infarction [23], stroke [23, 24], and hyperglycemic crisis [23]. Consequently, a delay in ED presentations has been shown, possibly negatively affecting the medical management of oncological patients [25]. Retrospective observational studies of the pandemic have demonstrated diminished abdominal surgical emergency admissions [20, 26], with abdominal conditions that presented having an increased severity based on clinical [15], radiological [20, 21, 26, 27], and pathological [22, 28] data. Based upon this research during the initial COVID-19 pandemic, it is plausible that people avoided the ED to a point where their condition progressed to become more serious, whereas pre-pandemic, these individuals would have sought healthcare earlier in their disease’s natural course.

The main objective of this study was to examine how the public-health lockdown impacted the EDs in two major hospitals within St. John’s, NL. A retrospective quantitative analysis was employed to examine the incidence of two common abdominal pathologies: diverticulitis and appendicitis, presenting to the ED prior to (January–February), during (March–June), and following (July–August) the COVID-19 lockdown. At each interval, we examined (1) the number of patients seen in the ED; (2) the amount of computed tomography (CT) abdominal scans ordered in the ED for abdominal pain; (3) the number of positive cases for diverticulitis or appendicitis; and (4) the subset of positive cases that were complicated. Despite NL being able to significantly reduce the number of COVID-19 cases within the province during the initial lockdown, we hypothesized that there would be a similar effect on ED visits, CT abdominal scans, positive rates, and severity of abdominal conditions, like others who have been previously mentioned. Specifically, we expect that the amount of CT abdominal scans ordered in the ED will be lower and that there will be more complicated cases of diverticulitis and appendicitis during the lockdown compared to previous years.

## Methods

**Fig. 1:**
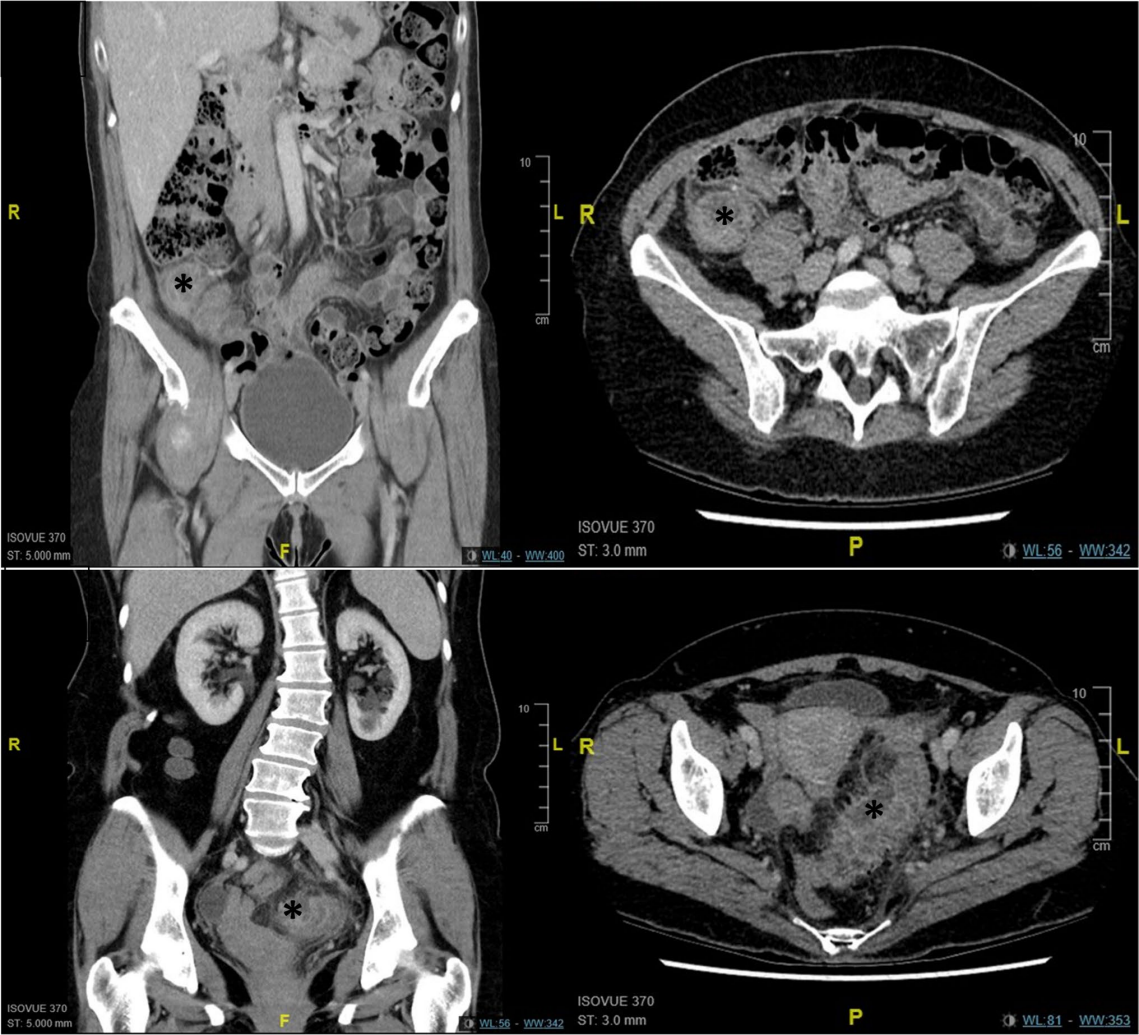
**A** Coronal and axial cut of a CT abdominal scan for patient with a complicated case of appendicitis with abscess demonstrated with an asterisk. **B** Coronal and axial cut of a CT abdominal scan for patient with a complicated diverticulitis with abscess demonstrated with an asterisk

A retrospective quantitative analysis gathered information on ED visits via the Newfoundland and Labrador Centre for Health Information (NLCHI). NLCHI compiled ED adult (19 +) visits and their demographics; age, sex, and Canadian Triage and Acuity Scale (CTAS) scores, from the major EDs in St. John’s, NL: (1) Health Sciences Center (HSC) and (2) St. Clare’s Mercy Hospital (SCM). A month-to-month comparison was performed to represent three different intervals: pre-lockdown (January–February), during lockdown (March–June), and post-lockdown (July–August) over three consecutive years 2018, 2019, and 2020. Only visits between January 1st and August 31st for the years 2018, 2019, and 2020 were obtained (Table 1). The years 2018 and 2019 were chosen as a control to compare our data from 2020, as these years did not experience a pandemic. Additionally, we included the months outside of lockdown as an added control to limit the possibility of other confounding variables (i.e. changes in healthcare emergency policies) that may have occurred in the initial months of 2020. A post-lockdown period was included to further understand the lockdown’s effects by examining for delayed presentations of complicated appendicitis/diverticulitis as the healthcare system recovered. *mPower* (Nuance Communications, UK), a software that extracts data from the Picture Archiving and Communication System (PACS) (a dedicated storage, retrieval, distribution, and display of diagnostic images), was utilized to collect the total volume of CT abdominal scans completed on patients presenting with abdominal pain. Appropriate CT abdominal scans ordered were collected by only searching ED physicians that worked during the years of 2018–2020 and by applying these specific key words: “Abdominal Pain”, “Epigastric Pain”, “Appendicitis”, “Right Lower Quadrant Pain”, “RLQ Pain”, “Diverticulitis”, “LLQ Pain”, “Left Lower Quadrant Pain”, “Right Upper Quadrant Pain”, “RUQ Pain”, “Periumbilical Pain”, “LUQ”, “Left Upper Quadrant Pain”. We then utilized PACs to examine radiology reports for each CT abdominal scan to categorize each diagnosis into one of three categories: (1) normal/other; (2) appendicitis; or (3) diverticulitis. Next, an experienced staff radiologist examined the radiology report for each case of appendicitis and diverticulitis to classify whether it was uncomplicated or complicated (i.e. perforation, abscess, obstruction, etc.) (Fig. 1).

**Table 1:** Demographics of patients presenting to the HSC and SCM emergency departments from January to August 2018–2020

Demographics variables, *n* (%)				
Total ED visits (*N*)	177133			
HSC	104200 (59)			
SCM	72933 (41)			
Gender, *n* (%)				
Male	80738 (46)			
Female	95606 (54)			
Undisclosed	0 (0)			
Median age, (IQR)	51 (33)			
Average age	67			
Age, *n* (%)				
19–39	59840 (34)			
40–59	52605 (30)			
60–79	50088 (28)			
80 +	14600 (8)			
Number of patients per month	2018	2019	2020	Total
Month of visit, *n* (%)				
January	7838 (4)	8007 (5)	7455 (4)	23300 (13)
February	7530 (4)	6930 (4)	7368 (4)	21828 (12)
March	8292 (5)	8357 (5)	5746 (3)	22395 (13)
April	7409 (4)	7703 (4)	3921 (2)	19033 (11)
May	7685 (4)	7783 (4)	5891 (3)	21359 (12)
June	7480 (4)	7434 (4)	6552 (4)	21466 (12)
July	8357 (5)	7821 (4)	7413 (4)	23591 (13)
August	8291 (5)	8154 (5)	7716 (4)	24161 (14)
Total patients per year, *n* (%)				
2018	62882 (35)			
2019	62189 (35)			
2020	52062 (29)

Monthly visitation and presentation data were normalized to a consistent 30-day month equivalent. Visual examination of the pre-COVID data suggested that while variation existed month-to-month, the overall ED cases appeared relatively stable without clear seasonal variation or an indication of a year-to-year trend within the collection period. Expected values for the year 2020 were generated against the average normalized monthly visitation rates, as well as the upper and lower 95% confidence interval boundaries based on the preceding years. Expected frequencies of age, gender, CTAS scores, CT abdominal scans, pathology, and outcomes were also generated from the average normalized data of the preceding years as well as the 95% confidence interval boundaries. Chi-squared goodness of fit was used to test for deviations from expected values generated from the 2018–2019 data. Significance was considered at *p* < 0.05, but a significant effect was required to show significance from the mean expected values as well as showing significance against the 95% CI boundaries, as the generated expected average carried some uncertainty based on variation in the 2018–2019 dataset. This additional threshold for significance was set to ensure any noted changes were well outside of the previous month’s variation. To optimize the effective range of chi-squared analyses, per-day normalized rates were compared except for low-frequency measures, such as CT abdominal scans or positive cases, where monthly rates were used to ensure expected occurrences exceeded minimum count thresholds.

## Results

Between January 1st, 2018, and August 31st, 2018, the HSC and SCM EDs saw 62,591 people (28,406 males and 34,185 females), conducted 1134 CT abdominal scans, and had 220 positive cases (91 appendicitis and 129 diverticulitis), with 42 (8 appendicitis and 34 diverticulitis) being complicated diagnoses. In January–August 2019, 61,777 people (27967 males and 33,810 females) were seen in the ED, and 1238 CT abdominal scans were administered. There were 218 positive cases (84 appendicitis and 134 diverticulitis), 47 of those being complicated cases (15 appendicitis and 32 diverticulitis). Finally, in January–August 2020, 51,976 people (24,365 males and 27611 females) were in the ED, and 1145 CT abdominal scans were completed, with 212 positive cases (106 appendicitis and 106 diverticulitis) and 39 complicated cases (19 appendicitis and 20 diverticulitis).

**Fig. 2:**
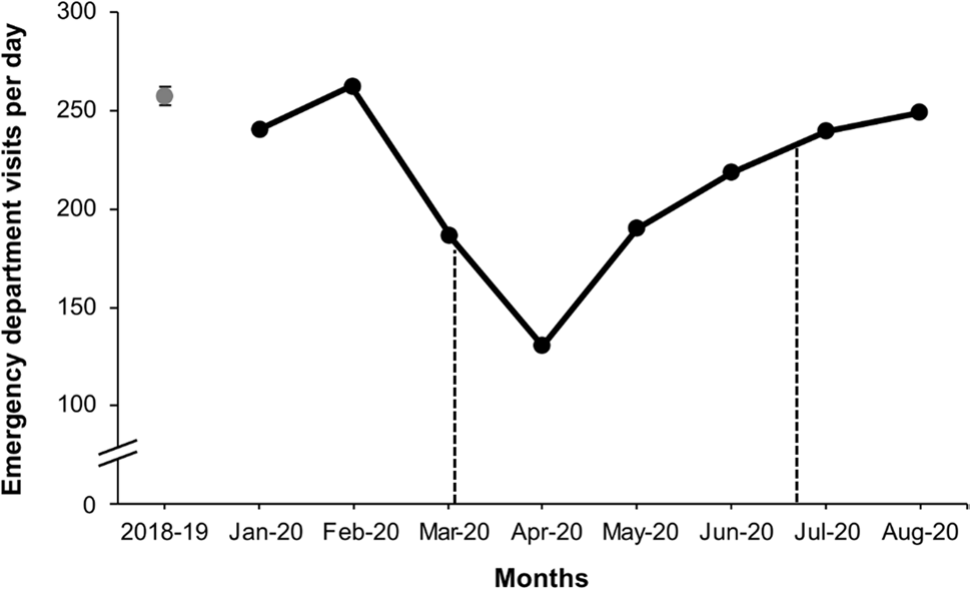
Significant decrease (*p* < 0.001) in number of observed ED visits per day for January to August 2020 (black dots) compared to expected (grey dot). The dotted black lines indicate the dates of the lockdown

The observed daily ED visits from January to August 2020 showed a significant (*p* < 0.001) reduction in total patient volumes from the expected daily ED visits. Specifically, 256 cases/day [95% CI 254–258] were expected, which we saw during pre-lockdown (Jan-Feb), while during lockdown (April 2020) this number dropped to an average of 131 cases/day (Fig. 2). This was independent of gender and age, with the observed daily ED visits in 2020 showing an equivalent range and variation as the daily expected ED visits in 2018–2019. There was no statistical difference between CTAS categories, with all showing a proportional reduction in the total number of patients presenting to the ED.

**Fig. 3:**
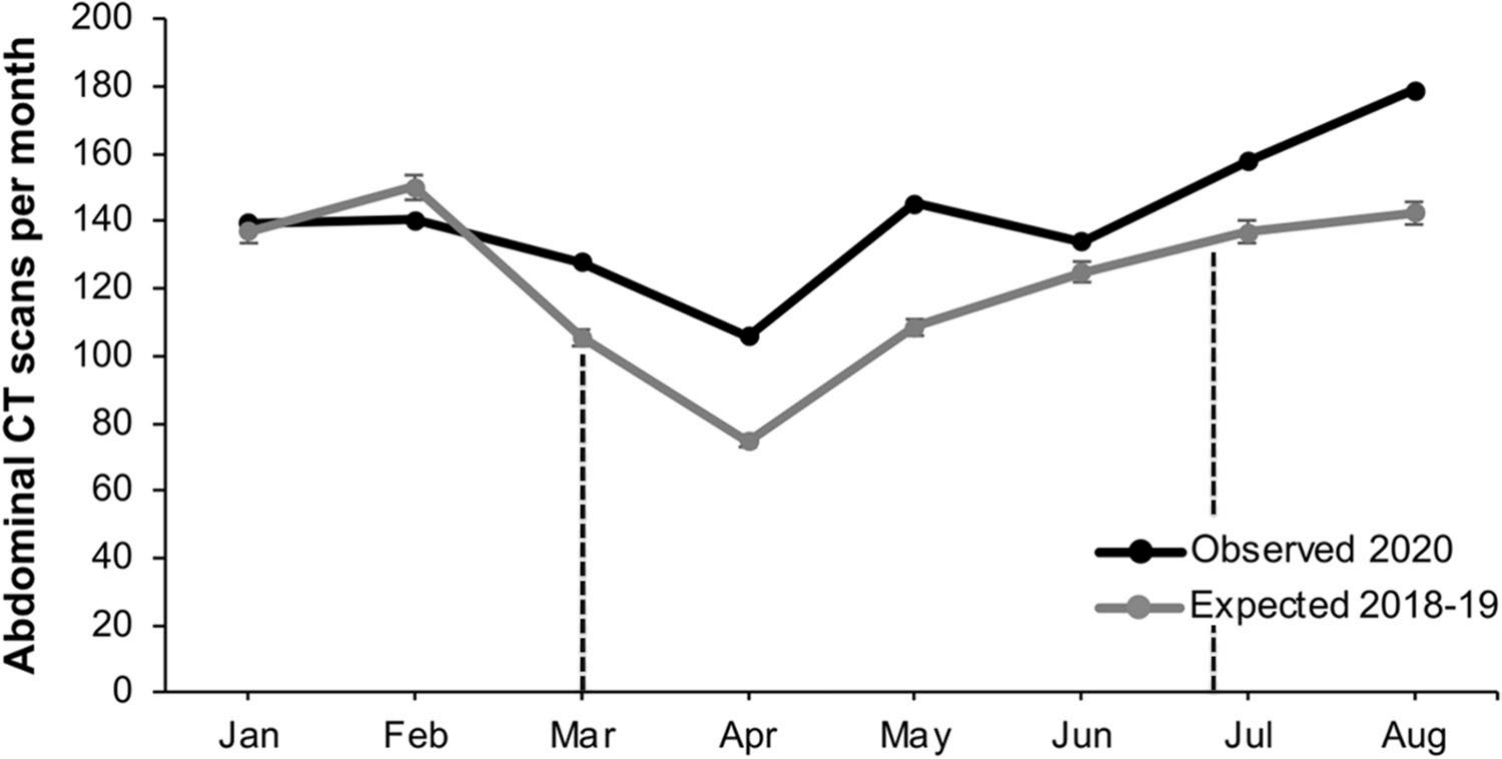
Significant increase (*p* < 0.001) in observed CT abdominal scans per month (black dots) compared to expected (grey dots). The dotted black lines indicate the dates of the lockdown

The CT abdominal scans ordered throughout 2020 were relatively consistent during pre-lockdown. There was a reduction during the lockdown, but it did not proportionally drop to the total reduction of patients presenting to the ED (*p* < 0.0001), indicating patients whose conditions would normally necessitate CT scans were still presenting to the ED and scans were ordered (Fig. 3). The post-lockdown period showed an increase in CT scans ordered.

**Fig. 4:**
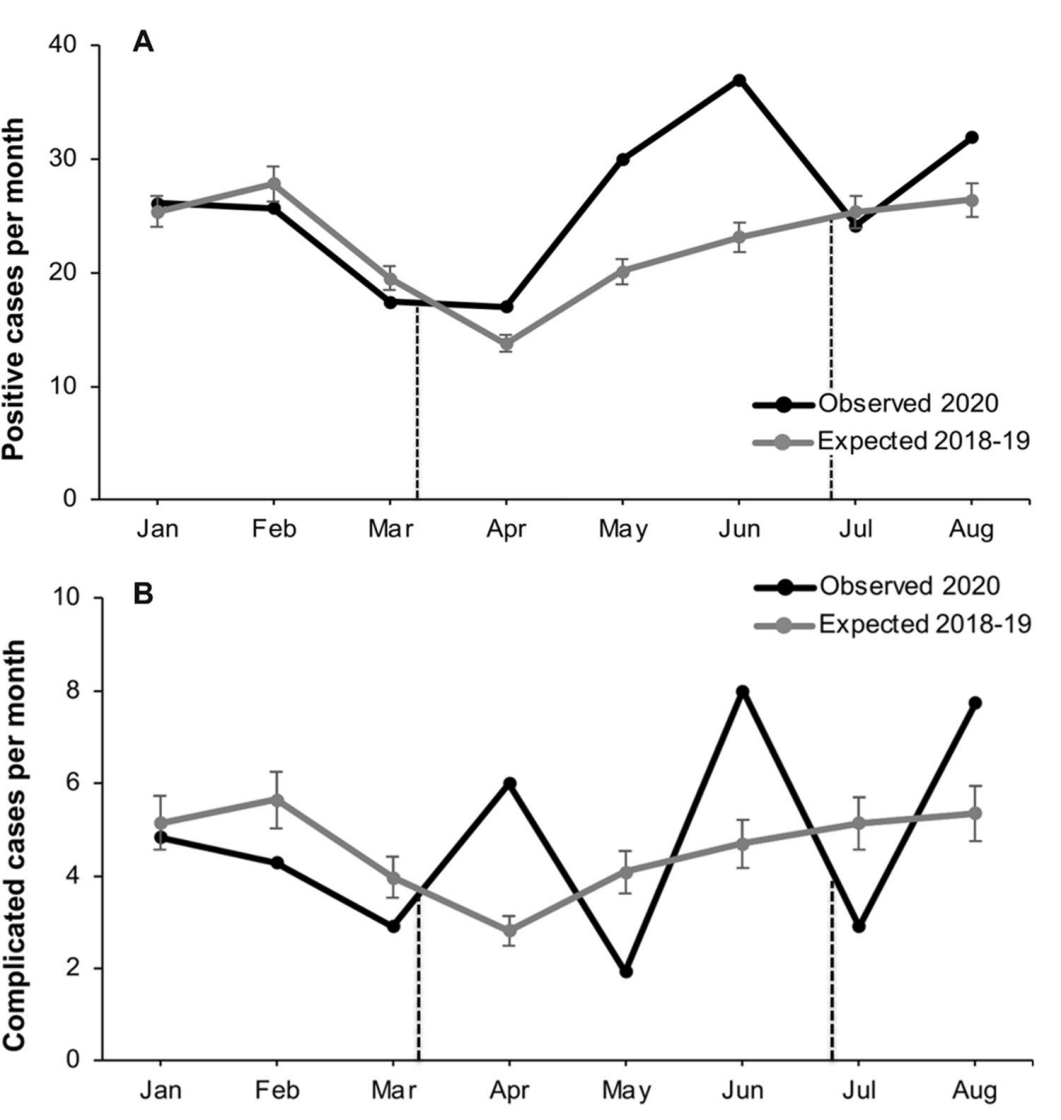
Significant increase (*p* < 0.05) in observed combined pathology (**A**) per month (black dots) compared to expected (grey dots). No significant difference was shown (*p* > 0.05) between observed combined complicated cases (**B**) per month (black dots) compared to expected (grey dots). The dotted black lines indicate the dates of the lockdown

The observed combined (appendicitis and diverticulitis) positivity rate (Fig. 4) showed no conceivable difference during pre-lockdown. During early lockdown, from March to April, a reduction in positive cases was detected, with a corresponding surge in positive cases detected in May and June (*p* < 0.05). However, the rate of complicated cases remained relatively low, if highly variable, without a statistical overabundance in the late to post-lockdown period. Examining positive cases separated into appendicitis and diverticulitis (Table 2) suggested more nuanced and complicated relationships. The total cases of appendicitis did not proportionally drop during the 2020 lockdown and showed significant divergence from the expected proportion of positive patients (*p* < 0.001). However, positive cases of diverticulitis did reduce throughout the lockdown and appeared proportional to the total number of patients presenting to the ED. Cases of diverticulitis returned to pre-COVID levels following the lockdown (*p* > 0.05).

**Table 2:** Observed (per month) appendicitis and diverticulitis cases and observed complicated appendicitis and diverticulitis cases compared to expected for January to August in EDs in St. John’s, NL. Significant *p* values are italicized and denoted with a *

Month of visit	Jan	Feb	Mar	Apr	May	Jun	Jul	Aug	*p*-value
Appendicitis									
Observed	13	9	12	10	17	23	8	14	< 0.0001*
Expected (mean)	10.2	11.1	7.8	5.5	8.1	9.3	10.1	10.6	
Expected (95% CI)	(9.2, 11.1)	(10.1, 12.2)	(7.1, 8.6)	(5, 6.1)	(7.3, 8.8)	(8.4, 10.1)	(9.2, 11.1)	(9.6, 11.5)	
Complicated									
Observed	2.9	1.1	2.9	2.0	1.0	4.0	1.0	3.9	0.067
Expected (mean)	1.3	1.4	1.0	0.7	1.0	1.2	1.3	1.4	
Expected (95% CI)	(1.1, 1.6)	(1.2, 1.7)	(0.8, 1.2)	(0.6, 0.9)	(0.8, 1.2)	(1, 1.4)	(1.1, 1.6)	(1.1, 1.6)	
Diverticulitis									
Observed	13.5	17.1	5.8	7.0	12.6	14.0	16.5	18.4	0.788
Expected (mean)	15.2	16.6	11.7	8.3	12.1	13.9	15.2	15.8	
Expected (95% CI)	(14.1, 16.3)	(15.4, 17.9)	(10.8, 12.6)	(7.7, 8.9)	(11.2, 13)	(12.8, 14.9)	(14, 16.3)	(14.6, 17)	
Complicated									
Observed	1.9	3.2	0.0	4.0	1.0	4.0	1.9	3.9	0.308
Expected (mean)	3.8	4.2	3.0	2.1	3.0	3.5	3.8	4.0	
Expected (95% CI)	(3.3, 4.3)	(3.6, 4.8)	(2.6, 3.4)	(1.8, 2.4)	(2.6, 3.5)	(3, 4)	(3.3, 4.3)	(3.4, 4.5)	

## Discussion

Our study is the first to examine how the COVID-19 lockdown affected EDs within NL. Overall, during the initial COVID-19 lockdown, from March to June, the EDs in St. John’s experienced a significant decrease in the number of patients seen. This decline was irrespective of both age and sex, as a similar decrease was seen across all demographics. Additionally for the 2020 timeframe, there was no change in the CTAS scores that presented to the ED, inferring no change in acuity seen during the lockdown. In contrast, the amount of CT abdominal scans ordered remained relatively consistent during lockdown and did not match the reduction in patient loads. Proportionally higher use of CT scans remained into post-lockdown, July, and August, when restrictions were eased, and patient numbers increased. An increase in the amount of observed combined positive cases was seen over the lockdown with no change in combined complexity rates. However, when examining appendicitis and diverticulitis independently, only observed positive appendicitis rates appeared indifferent to lockdown-related reduction in patient volume, whereas diverticulitis did reduce and returned in proportion to total patients presenting to the ED. This data suggests that despite the lockdown, cases of appendicitis still made their way to the ED and progressed as usual without an increase in complicated cases. The lockdown month of June did show an unusually high number of appendicitis-positive cases compared to previous years but did not have a preceding delay/drop in cases. Therefore, concerns about increased complicated appendicitis cases did not materialize. On the other hand, cases of diverticulitis did not surge, suggesting a backlog or delay of diagnosis was not occurring. Diverticulitis cases appear to show a relationship to lockdown conditions but not due to delays in avoiding ED and CT scans. The complexity rates of diverticulitis remained low and unaffected. The observed difference in the number of CT abdominal scans contradicts our hypothesis. While we predicted an increase in positivity rates, we did not anticipate appendicitis and diverticulitis diverging in their responses to COVID-19 lockdown. Attending physicians may have been reacting to a sense of caution or concern that complicated cases were unaddressed during lockdown, leading to higher CT scan orders post-lockdown, but that risk did not appear to materialize.

Across the world, the literature has consistently shown that the amount of people in EDs significantly decreased during early pandemics or respective lockdowns [14–22]. They have attributed this to several reasons, the most probable being fear of exposure to COVID-19 in the ED and simply following local lockdown recommendations [29, 30]. We found no effect of age or sex on visitation to the ED, with all demographics reacting equivalently. Janke et al. (2021) found that there was a greater decrease in individuals aged 75 years and older compared to the younger population across the United States of America (USA) [31]. In our study, while there was an incremental decrease across all age groups during lockdown, there was an subsequent equal resurgence back to baseline post-lockdown. One plausible reason for this discrepancy is the number of active cases that were present in NL compared to the USA. Specifically, NL had 261 total cases (0.05% of the population) over the full lockdown [13], while the USA had over 2 million (0.60% of the population) [32]. Thus, in NL there might have been less hesitation in traveling to an ED if one truly needed emergent care. This can be further justified by examining the CTAS scores as they showed no change in the acuity of patients that were seen in the ED over the lockdown period compared to our control years and pre-lockdown. With a lower amount of ED visits, one would expect the patients seen to be of higher number acuity, i.e. CTAS 1, 2, or 3. However, this was not the case instead, there was a drop off across all of the CTAS scores, indicating that the majority of people followed the rules and stayed away from the ED, but those who wanted to go were not discouraged by fear of catching COVID-19, even if their condition was not ruled an emergency (i.e. CTAS 5). However, even emergent, and critical CTAS caseloads dropped, suggesting the COVID-19 lockdown had indirect effects and may have reduced overall population emergencies brought on by sports, car accidents, or other activities stifled by lockdown.

The proportional number of CT abdominal scans is opposite of what other ED departments have reported across the world. For example, using machine learning with natural language processing, Li et al., (2021) examined radiology reports for CT abdomen/pelvis, CT abdomen, and CT pelvis from a Massachusetts hospital. They specifically looked for acute appendicitis, acute diverticulitis, and bowel obstruction from January 1st, 2018, to August 14th, 2020. Their results showed a significant decrease in both the volume of CT abdomen/pelvis scans as well as the detection of acute abdominal pathologies. However, they found that the number of scans conducted quickly rebounded back to the historical amount in the months of June and July [22]. This has been a consistent finding in multiple other studies [18, 19, 33–35], which have echoed substantial decreases in the amount of CT scans ordered early in the pandemic.

Within the literature, the findings on pathologies have been mixed. Several studies have shown patterns of decreased detection [15, 27], while others have shown increased positivity rates on appendicitis [20, 36]. For example, O'Brien et al. (2020) found an increased rate for both appendicitis and bowel obstruction, but a decrease in rate for diverticulitis and malignancy [19]. Our results recapitulate the finding of reduced diverticulitis during lockdown and imply behavioural intervention brought on during lockdowns may impact diverticulitis onset. Similarly, examining CT scans of the abdomen and pelvis for all abdominal complaints from two hospitals in Utah, Griffith et al. (2021) reported a 31.6% decrease in CT abdomen and pelvis scans in April 2020 compared to April 2019, while having a higher positivity rate in 2020. However, the only abdominal complaints that were significantly different in 2020 for positivity rate were appendicitis, cholangitis, and colitis, whereas the other 14 abdominal complaints showed no difference between the two timepoints [18].

Few studies have examined how COVID-19 affects the complexity of pathologies presenting to EDs. These studies have shown an increased complexity of pathologies, including appendicitis and bowel obstruction [19], appendicitis in children [36], appendicitis and cholecystitis in adults [28], and acute coronary syndrome [37]. This information indicates that in other hospitals, patients were likely hesitant to enter an ED when they had an acute medical condition. Therefore, instead of seeking medical attention immediately, they waited until their condition deteriorated to a problematic stage. On the contrary, our combined pathology data seems to represent the opposite, with patients who were actively ill proceeding to travel to the ED early in their disease course without hesitation.

From our knowledge, our study is the first to observe an increase in CT abdominal scans with an increase in combined positive rates but no change in complexity of the pathology. We believe the discrepancies shown in our data can be explained by a change in clinical practice due to several factors. It has been shown that in times of infectious outbreaks, physicians tend to change their behaviour and alter their clinical practice [38]. In the beginning of the pandemic there was very little known about COVID-19. The symptoms of COVID-19 were still novel; thus, it was unknown when and if a patient’s chief complaint of abdominal pain was a symptom of COVID-19. Recommendations were to investigate individuals with abdominal pain, and reduce the spread of COVID-19 [39]. Therefore, using a strong diagnostic tool like the CT scan would be one of the main triaging tools utilized to assess the patient for any serious medical ailments. The practical usage of the CT, with its ability to obtain a wealth of information in a quick, efficient, and non-invasive manner, makes it the ideal method for assessing, and understanding injuries or insults in patients [40]. Additionally, if we break down the pathologies independently into appendicitis and diverticulitis, we see unanticipated results. One would expect an increase in positivity rates for both pathologies, but instead, appendicitis rates continued unabated during lockdowns while diverticulitis rates fell proportionally to the total number of patients in the ED. Once again, we attribute these differences to a change in clinical practice of ED physicians. During times of normal clinical practice, the first-line diagnostic imaging test for a suspected appendicitis is the CT scan; however, ultrasonography (US) is considered an appropriate alternative depending on the clinical picture (i.e. symptoms, and age of the patient) [41]. While we did not look at the number of ultrasounds performed, we did observe that during the lockdown the number of CT abdominal scans and positive appendicitis cases did not fall in proportional to the total patients, which coincides with a relative increase in the usage of the CT abdominal scan being preferred. This pattern has been shown in two multicenter cohort studies examining appendicitis during COVID-19, both of which showed an increase in CT imaging and a decrease in the number of US [42, 43]. This further highlights the importance of imaging as an essential tool in a clinical scenario.

Another plausible factor attributing to our results is that at the start of the pandemic, NL implemented a virtual care code for family physicians, making primary care more accessible during the public health lockdown [44]. Patients that had diverticulitis previously may have sought their primary providers and received treatment, negating the need to travel to an ED. This would have a two-fold effect as it would decrease the patient load on the ED, and the outpatient care would avoid complicated cases. Without this change, we may have seen more diverticulitis cases within the ED. Finally, the method by which we represented our data is different in comparison to similar studies. We developed a control from the years 2018 and 2019, thus creating a predicted value that we should have observed in 2020 if there was no pandemic. Therefore, the predicted number represents patients that would require a CT abdominal scan if 2020 were a “normal” year, which our pre-lockdown data shows that before COVID-19 it was trending as equivalent. Other papers chose to present their data using different methods, such as the total percent change in patients seen and CT abdominal scans ordered [18, 19, 22, 33–35]. Consequently, the relative increases in the amount of CT abdominal scans shown in our study might have been equivalents at other centers if their data were shown as compared to normalized trends.

The limitations of our project are as follows: first, it is possible that other EDs across the province did not experience the same patterns of patient presentations as the two hospitals in St. John’s. Therefore, our generalization of what occurred may not be accurate for the entire province. Additionally, we have no concrete reasoning as to why rates of appendicitis and diverticulitis behaved so differently during lockdown. It is possible that US (diagnostic or POCUS) and other types of imaging modalities were also significantly changed during lockdown and post-lockdown, and we can only speculate as to what factors may have caused this outcome. Local future studies would benefit from investigating how the lockdown affected other medical conditions, imaging modalities, and physician’s clinical practices.

In conclusion, our study has shown that during the initial COVID-19 lockdown, the two EDs within St. John’s, NL saw a significant decrease in the number of patients seen during lockdown. The number of CT abdominal scans ordered and pathology diagnosed, specifically appendicitis cases, were higher then expected, which remained consistent with non-COVID practice. This data shows that the people of NL did not withhold themselves from seeking medical attention when necessary, while most people who did not need to seek medical attention remained home. Additionally, the usage of imaging as a diagnostic modality is crucial in times of uncertainty and should be utilized as an important tool in diagnosing and triaging patients in the ED.
